# *Mycoplasma leachii* sp. nov. in Calves, China

**DOI:** 10.3201/eid1709.101891

**Published:** 2011-09

**Authors:** Ji-Tao Chang, Hai-Jun Liu, Li Yu

**Affiliations:** Author affiliation: Harbin Veterinary Research Institute–Chinese Academy of Agricultural Sciences, Harbin, People’s Republic of China

**Keywords:** bacteria, cattle, calves, mycoplasma, Mycoplasma leachii, arthritis, China, letter

**To the Editor:** *Mycoplasma leachii* sp. nov., a new species designation for *Mycoplasma* sp. bovine group 7 (*1*), was initially isolated from joint fluids of arthritic calves in southern Queensland, Australia, and its pathogenicity was established by experimental infection (*2*). It was represented by the type strain PG50. Subsequently, *M. leachii* was reported infrequently as a cause of polyarthritis in calves and mastitis in cows; the pathogen was also isolated from aborted fetuses and pneumonic bovine lungs (*3**–**6*) and from small ruminant hosts (*7*).

*M. leachii* is one of 5 recognized members of the *M. mycoides* cluster, which comprises 3 species (*1*). Most notable are *M. mycoides* subsp. *mycoides* small colony and *M. capricolum* subsp. *capripneumoniae*, the etiologic agents of contagious bovine and caprine pleuropneumonia, which are listed by the World Organisation for Animal Health as notifiable animal diseases. The *M. mycoides* subsp. *capri* and *M. capricolum* subsp. *capricolum* cause various symptoms in small ruminants (*8*). Strains of *M. leachii* that cause mastitis and polyarthritis in cattle are serologically distinct from other bovine *Mycoplasma* spp (*9*). Most reported isolates of *M. leachii* were detected in Australia. We report the isolation of *M. leachii* in cattle in China.

During January–May 2009, severe polyarthritis was observed in ≈100% of ≈350 female calves at the central calf rearing unit of a farm in Helongjiang Province, People’s Republic of China. Clinical signs were noticed at ≈3–5 days of age, with severity gradually increasing over the next 2 days. At that time, the carpal and tarsal joints were greatly enlarged because of accumulation of intraarticular fluid. Ampicillin, sulfonamide, and streptomycin antimicrobial drug regimens for polyarthritis were ineffective. Approximately 100 calves died during the outbreak; the remaining calves recovered irrespective of treatment, but permanent disfigurement of the appendicular skeleton was evident. The disfigurement led to the calves being culled.

Necropsy was conducted on the calves that died during the outbreak, and gross and histopathologic findings similar to those described (*2**,**3*) were observed. Nearly all diarthroidal joints were enlarged and contained yellow-gray turbid synovial fluid and large yellow fibrin clots. The synovial membranes were slightly thickened, congested, and had some villous proliferation. Histologic examination of the affected articulations found severe, diffuse, subacute arthrosynovitis and bursitis.

Routine bacterial culture of 2 joint fluid samples collected aseptically from different animals showed no bacterial growth. *Mycoplasma* spp. infection was suspected, and the joint fluid samples were forwarded to the laboratory for specific culture; 2 were positive for *Mycoplasma* spp*.* These isolates were designated GN407 and GN408.

The presence of *M. leachii* in joint fluids and *Mycoplasma* spp.–positive cultures was detected by PCR with the partial *lppA* gene amplified with a protocol modified from the method described by Frey et al. (*10*) and amplification of the complete 16S rRNA gene was performed by using the primers 16S-upper 5′-AAAATGAGAGTTTGATCCTGG-3′ and 16S-lower 5′-AGAAAGGAGGTGATCCATCCG-3′. The primers were designed on the basis of the 16S rRNA gene sequence of *M. leachii* PG50 (U26054). PCR products were sequenced directly in both directions. Sequence analyses were conducted by using MEGA version 4.1 (www.megasoftware.net). The partial *lppA* gene nucleotide sequences of isolates GN407 and GN408 were submitted to GenBank under accession nos. HQ699892 and HQ699893, respectively.

PCR amplifications of the 2 joint fluids and their cultures were positive for *M. leachii*. When we compared the complete 16S rRNA gene and the partial *lppA* gene, the 2 isolates from the same epizootic shared 100% nt identity. For 16S rRNA gene, the isolates shared 99.9%, 99.9%, and 99.7% nt identities to *M. leachii* PG50, *M. capricolum* subsp. *capricolum*, and *M. mycoides* subsp. *mycoides* small colony, respectively. For partial *lppA* gene, the isolates shared 99.6%, 95.1%, and 69.6% nt identities to *M. leachii* PG50, *M. mycoides* subsp. *mycoides* small colony, and *M. capricolum* subsp. *capricolum*, respectively.

Intraarticular inoculation of the passage cultures successfully reproduced the polyarthritis in calves 1 month of age. Thus, there are notable similarities between our findings and those reported in Australia (*3*). Multidisciplinary procedures, including clinical assessment and comprehensive laboratory investigations of affected calves, were used to identify the etiologic agent. The results showed that the outbreak of the serious polyarthritis in calves was caused by *M. leachii*.

Our detection of *M. leachii* in China confirms a wider geographic presence of this type of *Mycoplasma* spp. in cattle and suggests *M. leachii* is common and potentially distributed worldwide. Currently, the source of *M. leachii* infection and its means of spread have not been established. However, our epidemiologic and clinical investigations indicated clear evidence of seminal infection because all calves with arthritis were from dams that were fertilized by using the same batch of semen, and cows in the same herd that were fertilized by using a different batch of semen delivered healthy calves. More epidemiologic, molecular, and pathogenic studies are required to determine the relevance, distribution, importance, and diversity of *M. leachii* in cattle.
